# Decentralized Proof-of-Location systems for trust, scalability, and privacy in digital societies

**DOI:** 10.1038/s41598-025-04566-4

**Published:** 2025-06-05

**Authors:** Eduardo Brito, Amnir Hadachi, Liina Kamm, Ulrich Norbisrath

**Affiliations:** 1 Cybernetica AS, Narva mnt 20, Tartu, 51009 Estonia; 2 University of Tartu, Narva mnt 18, Tartu, 51009 Estonia

**Keywords:** Proof-of-Location, Decentralized Systems, Trustworthy Location Verification, Computer science, Information technology

## Abstract

Verifying physical presence in digital systems is essential for secure authentication, authorization, and accountability. Proof-of-Location (PoL) systems address this need by enabling verifiable, tamper-resistant claims of location and time, particularly in adversarial environments where traditional localization methods such as GPS fall short. While recent efforts have explored decentralized PoL architectures, existing systems often lack a unified model that integrates spatio-temporal synchronization, distributed consensus, and cryptographic attestation. In this paper, we formalize the architectural foundations of decentralized PoL systems by introducing a composable model based on fault-tolerant witnessing zones. We define core components for synchronization and collective attestation, integrating primitives such as distributed digital signatures, distance bounding protocols, and consensus mechanisms. We contextualize the model across diverse application domains that necessitate digital trust-such as civic processes, content authentication, infrastructure auditing, and supply chain tracking-and argue for a shared, interoperable decentralized PoL infrastructure. To validate our design, we implement and emulate a reference protocol instance, analysing scalability, synchronization behaviour, and timing misalignment under varied conditions. We also outline its security model and discuss limitations and future directions in privacy, interoperability, and real-world deployment. Our contributions lay a formal and practical foundation for scalable, secure, and general-purpose decentralized PoL systems.

## Introduction

Ensuring trust in location data remains a critical challenge in digital systems. While modern localization technologies provide precise positioning, they lack mechanisms to guarantee the authenticity and integrity of location claims^1,2^. GPS and similar systems, though widely used, are vulnerable to spoofing and tampering and do not offer cryptographic assurances of a device’s presence at a specific location and time^3^. As a result, they are unsuitable for applications requiring verifiable and tamper-resistant location proofs, particularly in adversarial settings. The absence of a spatio-temporally sound system remains a barrier to realizing the full potential of secure location-based services and applications^4^. Addressing this challenge requires synchronization and cryptographic attestation mechanisms capable of operating under partial trust. Establishing location as a verifiable digital primitive could enable new forms of secure authentication, authorization, and accountability in digital infrastructures^5^.

Proof-of-Location (PoL) systems address this need by enabling the generation of verifiable claims of presence at a specific location and time. Such systems are foundational for applications that require location-bound authentication or authorization, including customer reward programs, liability-based services such as delivery or access verification, and the protection of location-sensitive digital content^6^. Furthermore, the rise of generative AI has underscored the need to certify the originality and physical authenticity of digital content^7^, making even more critical tamper-proof PoL solutions for certifying the physical provenance of digital content. Recent research has explored PoL designs across a spectrum of trust models, ranging from centralized systems with strong assumptions to emerging decentralized architectures designed for adversarial and permissionless environments^8^.

### Problem statement

Witnessing is a fundamental component of location proofs, establishing trust through the testimony of one or more parties who verify a claimant’s presence at a specific place and time. At its core, witnessing relies on the spatio-temporal coexistence of entities with distinct roles. Central to location attestation is the notion of simultaneity-the occurrence of events at the same time. In this context, simultaneity extends to both space and time as the approximation of shared presence, or spatio-temporal coincidence^9^. For a location claim to be valid, entities must be close in space and agree on the time of the claim. This dual requirement introduces the need for both spatial constraints on communication and temporal synchronization among participants.

**Fig. 1:**
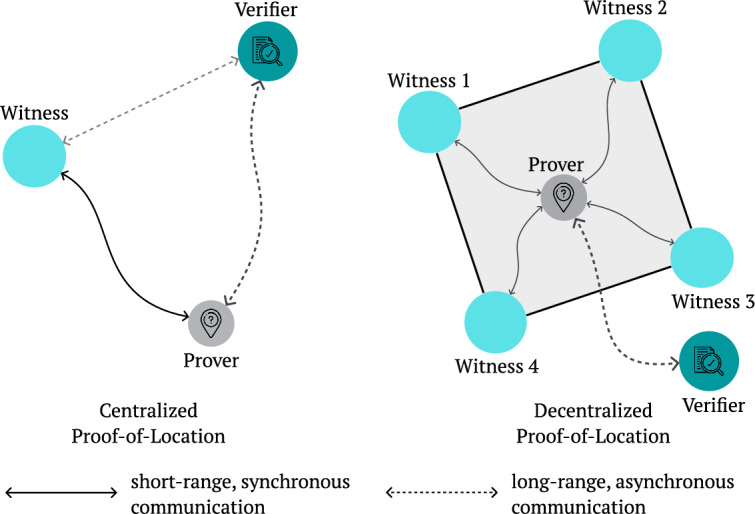
The entities involved in a PoL protocol. The left side of the figure illustrates a standard centralized protocol setup, in which the witness and verifier can establish a direct trust relationship. The right side shows the configuration of a decentralized solution, where a quorum of witnesses attests the prover’s location.

In brief, location proof protocols assume the existence of a *prover* who interacts with nearby *witnesses* to gather a verifiable PoL claim, which can later be presented to a *verifier* as evidence of the prover’s presence at a specific location and time^10^ (see Fig. 1). The spatial constraints are typically addressed through distance-bounded communication, ensuring witnesses can only attest to a location claim if the prover is physically nearby. The temporal aspect is equally critical, requiring mechanisms for relative clock synchronization^11^, so all parties agree on when the interaction occurred. Without this agreement, location proofs may be disputed due to inconsistent temporal records^12^.

In digital systems, this challenge then translates to the need for a robust protocol that can establish shared spatial and temporal understanding among a group of potentially untrusted but physically co-located entities. A digital PoL can thus be defined as an electronic certificate that attests the relative presence of a prover in both space and time^13^.

Inspired by^10,14^, we now introduce a general definition of the PoL problem, along with some of its desirable properties:

**Proof-of-Location.** A Proof-of-Location is a verifiable digital certificate that attests the presence of a prover *p* at location *l* and time *t*, by a set of witnesses $w \in W$.

**Spatio-temporal soundness.** A Proof-of-Location is spatio-temporally sound if it is generally hard for the prover *p* to obtain, forge, or modify a Proof-of-Location, if not physically present around a set of witnesses $w \in W$, at location *l* and time *t*.

**Completeness.** A Proof-of-Location generated by an honest prover *p* in cooperation with honest witnesses $w \in W$ can convince an honest verifier *v* of its truthfulness.

Additional properties, such as correctness, non-transferability, or privacy, may be protocol specific, but the above definitions are generally considered to be the most common and desirable properties of a PoL protocol. Further formalizations, including threat models for specific scenarios, can be found in the works listed in the next section.

### Related work

The need for systems to attest a device’s location arose early in the 21st century. Waters and Felten^1^ introduced a proximity-based model using a trusted setup involving a *verifier* and a tamper-resistant *device*, addressing use cases such as enforcing boundary restrictions for borrowed equipment. Their model combined wireless networks with PKI and proposed triangulation via multiple location managers to improve trust and precision. Saroiu and Wolman^6^ expanded the use cases to include customer rewards, location-restricted content, and voter verification. SLVPGP^15^ removed the need for a trusted location manager by shifting trust to tamper-resistant modules. VeriPlace^16^ distributed responsibilities across multiple trusted entities to reduce reliance on hardware and support public systems like Yelp. Javali et al.^17^ utilized existing Wi-Fi infrastructure to streamline proof generation, while Akand et al.^3^ addressed geo-tampering with provable security in centralized deployments.

As limitations of centralized systems became apparent, distributed models emerged to improve scalability, resilience, and privacy. APPLAUS^18^ combined proximity-based proofs with pseudonymous witnesses, using an untrusted location server to store anonymized claims. STAMP^19^ and PROPS^20^ introduced group signatures to enhance privacy and enable composite location proofs, though they struggled with collusion among participants. SPARSE^21^ reduced collusion risk by removing the prover’s control over witness selection and leveraging random crowded settings. Dupin et al.^10^ used secure multi-party computation (MPC) to preserve privacy during proof generation, at the cost of significant computational overhead^8^. These efforts reflect a clear shift toward decentralization, though many remained limited by coordination, collusion, and scalability challenges.

The advent of blockchain technology enabled fully decentralized PoL systems by eliminating centralized trust and storage. Amoretti et al.^13^ and Nasrulin et al.^14^ proposed peer-to-peer protocols leveraging blockchain for tamper resistance and open consensus. Wu et al.^22^ introduced zero-knowledge proofs (ZKPs) for selective disclosure in location proofs. Nosouhi et al.^23^ incorporated incentive mechanisms to promote honest participation and deter adversarial behavior. FOAM^24^ conceptualized a state-of-the-art decentralized system using time synchronization and LPWAN radio beacons, backed by Ethereum smart contracts. It introduced *Zone Anchors* and *Zone Authorities* to enable verifiable location claims while incentivizing infrastructure maintenance.

Despite these advances, a critical gap remains in formalizing the architectural components needed for scalable, interoperable decentralized PoL systems. Prior work has addressed individual aspects-such as spatial or temporal synchronization, secure localization, or consensus-but these contributions remain fragmented.

### Decentralization challenges and our contribution

While the concept of location attestation is common to many PoL systems, traditional implementations have often relied on centralized architectures or trust models that depend on trusted hardware or verifier-controlled infrastructure^1,16,18^. These models, though functional in controlled environments, face well-known challenges in terms of scalability, resilience, and cost when deployed across diverse real-world settings.

Prior research has typically focused on proof generation mechanisms or assumed pre-established trust relationships, often neglecting the complexity of decentralized environments. The need for decentralization becomes evident when considering practical limitations: verifiers may lack infrastructure coverage; provers may be unwilling or unable to interface with centralized services; and both may face risks of collusion or fragmentation across incompatible systems^6,20^. These constraints call for a new approach-one that enables secure, peer-to-peer generation and validation of location proofs without relying on trusted hardware, central authorities, or application-specific infrastructure.

Moreover, many PoL applications may coexist in overlapping physical environments. Redundant, siloed deployments not only waste resources but also increase complexity and hinder interoperability. A unified, decentralized PoL infrastructure would enable secure, verifiable location attestation across a wide range of use cases, maximizing efficiency and minimizing redundancy^24^.

While decentralized PoL systems have recently gained attention, significant gaps remain, particularly in their architectural formalization and compositional integration. In this work, we address that gap by formalizing the architectural components of a decentralized PoL system. Our model unifies previously scattered techniques-threshold cryptography, distance bounding, and spatio-temporal synchronization-into a composable, end-to-end framework.

We further ground this architecture in realistic use cases, including civic participation, supply chain verification, and digital content authentication. Based on these applications and prior research, we identify the minimal structural elements required to build scalable, interoperable decentralized PoL systems. Finally, we validate the proposed architecture through emulation of a single-zone protocol instance. We analyse scalability, timing, and synchronization dynamics under various deployment conditions, and outline directions for protocol instantiations, security analysis, and real-world deployment.

## Results

### Motivating PoL application scenarios

**Fig. 2:**
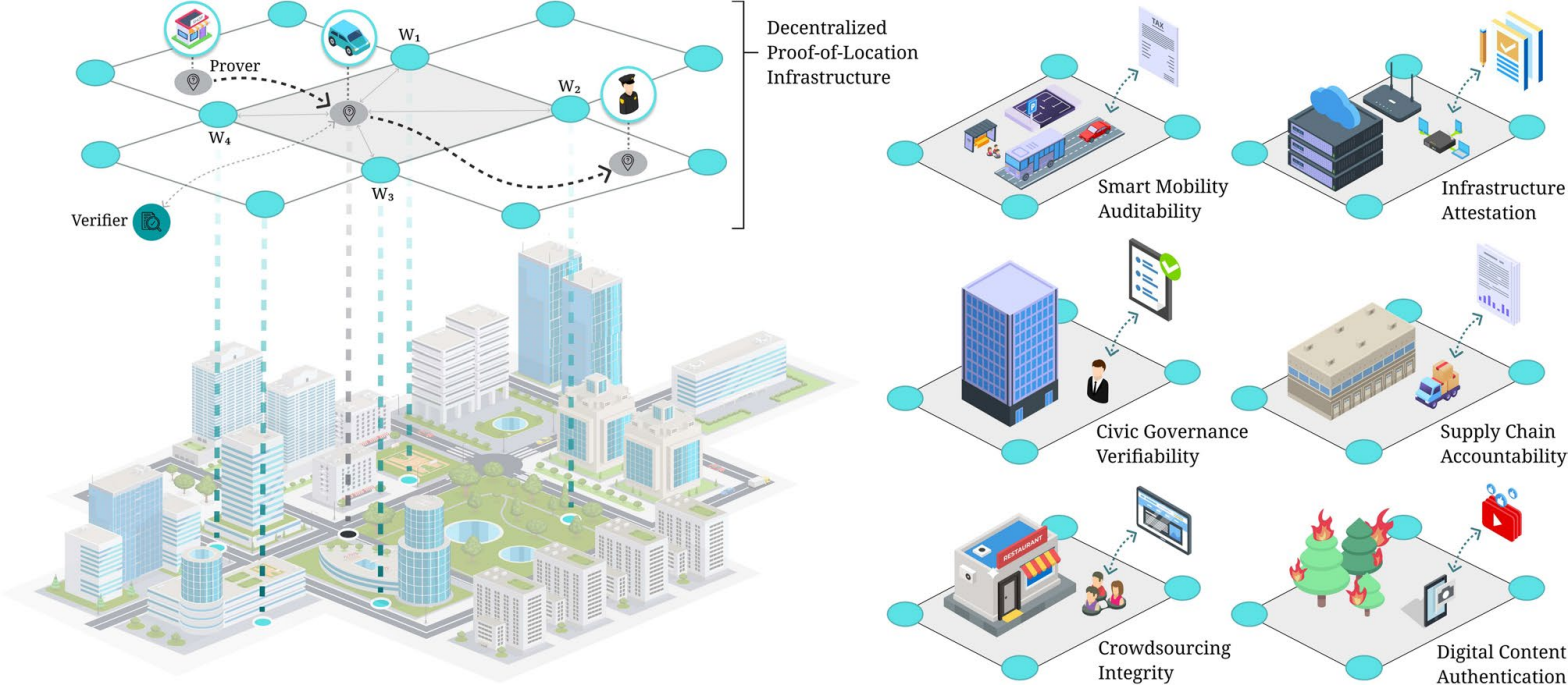
Decentralized Proof-of-Location (PoL) infrastructure across an urban environment. Provers interact with witnesses ($w_1$, $w_2$, etc.) to generate verifiable location proofs, which are later validated by verifiers. Diverse application sectors can coexist within the same physical environment and reuse the shared witnessing layer.

Building on the concept of a unified decentralized PoL system^24^, we first survey the broad landscape of applications for verifiable digital location proofs. These span commercial, civic, and technological domains^1,6,14,16,24–26^, highlighting the potential of a shared, secure infrastructure to support diverse use cases while reducing redundancy and improving efficiency across sectors. Figure 2 illustrates a conceptual overlay platform where multiple PoL applications coexist in the same urban environment by reusing a common witness infrastructure.

In the commercial sector, PoL can enhance customer engagement by enabling location-based loyalty programs and verifiable visit-based incentives^6^. In business review platforms, it can authenticate customer feedback by verifying physical presence, reducing fake reviews and improving the reliability of crowd-sourced information^16^. PoL also applies to content delivery, where it can enforce geofencing for services like video streaming, ensuring compliance with regional licensing agreements^1,6^.

The importance of PoL is further underscored by the rise of generative AI tools capable of producing highly realistic content^27^. In this context, PoL can help authenticate non-AI-generated media. Photographers, journalists, and other content creators can use PoL protocols to certify the physical provenance of digital assets, anchoring them to a specific location and time. This is particularly relevant for maintaining the credibility of news content in an era of digital manipulation^28,29^.

PoL also has practical value in supply chains, delivery services, and infrastructure deployment. Logistics providers can use it to verify package pickups and drop-offs, including those performed autonomously, enhancing transparency and reducing fraud^21^. Real-time integration with tracking systems ensures end-to-end authenticity and accountability. In customer-facing services, PoL can support dispute resolution and improve trust with attestable proofs of delivery.

For physical infrastructure and IoT deployments, PoL offers benefits across sectors. It enables authenticated verification of device installation and operational status-critical for environmental monitoring, smart cities, and industrial systems^22^. Telecom operators and ISPs can use it to verify infrastructure placement for regulatory compliance and service validation. In smart grid systems, it supports authenticated placement of energy infrastructure, enabling location-based management programs. PoL can also verify temporary deployments, such as in disaster recovery scenarios^30^. By providing reliable attestation of physical presence, PoL enhances transparency, service quality, and trust across both public and private domains.

In public transportation and urban planning, PoL can strengthen authenticity, liability attribution, access control, and accountability^26,31^. Cryptographic PoL attestations can underpin secure, fraud-resistant ticketing systems, reducing fare evasion and unauthorized access. They can also provide immutable records of passenger presence, aiding liability assignment in accidents or disputes. From an operational perspective, PoL enables transit agencies to demonstrate compliance with service-level agreements, such as schedule adherence or route coverage. It also supports fine-grained authorization for special services, like reduced fares for students or seniors, using verifiable, location-linked credentials. Aggregated, anonymized PoL data may offer urban planners actionable insights into infrastructure needs, promoting transparency in public investment^25^.

Beyond static environments, decentralized PoL infrastructures may also serve as trust layers in emerging dynamic systems such as vehicular networks, UAV-based operations, and mobile federated learning. By anchoring decisions in cryptographically verified spatial contexts, PoL systems can support secure peer evaluation, adaptive control, and resilient message dissemination ^32–34^. This approach enhances the integrity of mobility systems through distributed, data-driven verification.

In civic and governance applications, PoL can help authenticate voter presence at polling stations, reducing fraud and reinforcing electoral integrity. It may also support broader models of augmented democracy, enabling citizens to verifiably register attendance at public events and urban planning forums^25^. In public service delivery, PoL can simplify residency verification for taxation, registration, or access to location-specific services. Governments may also leverage PoL to better target subsidies and environmental incentives. For instance, authenticated presence in certain regions could trigger energy subsidies or location-based tax benefits for individuals or businesses. Likewise, PoL could support compliance with zoning rules or access restrictions in low-emission or car-free zones. By grounding policy enforcement in verifiable data, PoL improves governance efficiency, supports sustainability, and reduces reliance on self-reported or unverifiable claims^35,36^.

To support the diverse range of concurrent use cases, a secure and decentralized PoL system must coordinate both spatial and temporal synchronization among participants. The next sections define the core components required to construct a latticework of synchronized witnesses capable of collectively attesting to location proofs. In our model, individual witnesses need not be trusted by the prover or verifier; instead, trust emerges from the collective agreement of a fault-tolerant set within a zone. A prover generates a location proof, a verifier validates it, and both rely on the consensus of independent witnesses. This structure ensures a baseline of spatio-temporal soundness, while domain-specific applications may define additional validity criteria.

As PoL systems evolve, it is also critical to address privacy and ethical considerations. While PoL protocols aim to prove location at specific moments-not to track individuals-location data remains sensitive and must be protected. The goal is to offer verifiable proofs without enabling surveillance or compromising personal privacy. Future research should focus on privacy-preserving PoL protocols that provide selective disclosure, allowing users to prove presence without exposing unnecessary information or creating persistent traces. In parallel, legal and ethical frameworks are needed to govern these technologies. Such frameworks should prioritize data minimization, purpose limitation, user consent, and the protection of privacy in public spaces, helping ensure that PoL systems benefit society while upholding trust and individual rights.

### Witness latticework structure

**Fig. 3:**
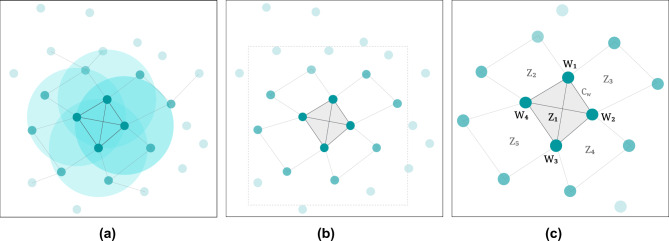
Witness latticework structure and zone establishment via a fully connected mesh network topology - (**a**) the physical distribution of witnesses in a 2-dimensional space and their communication ranges, (**b**) the discovery of zones, and (**c**) affinity of witnesses to multiple zones.

The system builds upon an *N*-dimensional spatial structure of interconnected witnesses that collectively form a dynamic latticework across space and time^24^. Each witness $w_i$ in the network is characterized by its position in physical space, defined by a generic set of coordinates $(x_i, y_i, z_i)$, and its zone-relative temporal state $t_i$. Within the network, witnesses maintain many-to-many peer-to-peer links, forming a dynamic and non-hierarchical fully connected mesh network topology^37,38^ (see Fig. 3) through two distinct communication channels, $C_w$ and $C_p$:

$$\begin{aligned} C_w= & \{(w_i, w_j) \mid w_i,\,w_j\text { are witnesses}\} \\ C_p= & \{(w_i, p) \mid w_i\text { is a witness},~p\text { is a prover}\} \ , \end{aligned}$$

where $C_w$ represents the witness-to-witness communication channel used for zone maintenance, synchronization, and consensus, and $C_p$ represents the witness-to-prover communication channel used for location attestation and proof generation.

A zone $Z_z$ represents a region of space-time where witnesses can reliably communicate and synchronize with each other, formally defined as:

$$\begin{aligned} Z_z = \{w_i \mid \forall w_j \in Z_z: (w_i, w_j) \in C_w \wedge d(w_i, w_j) \le R_{\text {max}} \wedge |t_i - t_j| \le \epsilon \} \ , \end{aligned}$$

where $d(w_i, w_j)$ represents the Euclidean distance between witnesses $w_i$ and $w_j$, $R_{\text {max}}$ is the maximum reliable communication range, and $\epsilon$ is the bound on the logical temporal precision between witnesses that is acceptable for coherent location attestations within the zone. Zones are defined globally, therefore, each $Z_z$ emerges as a cohesive subset of witnesses that satisfy mutual spatial and temporal constraints. This is not a witness-centric or recursive construction-zones are not initiated or owned by any single witness. The index *z* simply enumerates the different such zones in the network, and throughout the paper, we use *z* as a symbolic reference to a generic zone $Z_z$. The precision of the location proof is influenced by both the temporal bound $\epsilon$ and the spatial discretization range defined by the zone’s physical boundaries. A smaller zone area, controlled by a reduced $R_{\text {max}}$, allows finer spatial precision in the location proof, as it restricts the area within which a prover’s presence can be attested. However, this also comes at the expense of reduced coverage, as smaller zones cover limited physical space, potentially requiring more zones to maintain broader coverage across the latticework. Let $\mathscr {Z} = \{Z_1, Z_2, ..., Z_n\}$ be the set of all zones in the network. A witness $w_i$ may belong to multiple zones simultaneously, defined by the mapping:

$$\begin{aligned} M(w_i) = \{Z_z \in \mathscr {Z} \mid w_i \in Z_z\} \ . \end{aligned}$$

This multi-zone membership enables the formation of a continuous latticework structure, where zones overlap and interconnect through shared witnesses. The combination of multi-zone membership and overlapping zones creates an adaptable latticework structure. This structure can ensure continuous coverage across space while maintaining the necessary redundancy for reliable location attestation. Within each zone, the spatial, temporal, and connectivity constraints create an *N*-dimensional witnessing structure, where each zone constrains its space-time synchronization. Here, *N*-dimensionality refers to the spatial dimensions of the zone (e.g. 2D or 3D), clarified further in the next subsection. These zones may overlap, creating a dense latticework that can accommodate varying witness densities and adapt to dynamic network conditions. The separation of communication channels ($C_w$ and $C_p$) ensures that witness coordination and prover attestation can occur independently and securely. This structure provides the foundation for verifiable location attestations, as any prover seeking to generate a location proof must interact with witnesses within a given zone in order to obtain a valid attestation. The complete realization of a witness latticework requires the study of zone overlap dynamics, witness zone membership management, and inter-zone coordination protocols. This paper focuses only on the fundamental building blocks of a single zone configuration. Nonetheless, the establishment of secure and verifiable location proofs within a single zone serves as the foundation for future work on scaling and realizing a full latticework structure.

### Zone synchronization

The establishment of a synchronized zone is fundamental for verifiable location proofs, enabling the spatio-temporal soundness and completeness demanded by a PoL protocol. While spatial synchronization among witnesses is implicitly enforced by physical communication range constraints and a non-hierarchical fully connected mesh network topology, temporal synchronization requires an agreement on event order and timing. Consensus is the process by which distributed entities reach a common state or ordering, even if some parties fail or act maliciously. In this context, witnesses must collectively attest to a prover’s presence at a single, agreed-upon moment in time. This alignment of clocks and event ordering can be seen as a specialized form of the distributed consensus problem, requiring a fault-tolerant coordination strategy to mitigate adversarial behaviour. Ultimately, such synchronization and final ordering of recorded events can be provided by a Byzantine fault-tolerant consensus mechanism that guarantees deterministic finality for a finite set of witnesses^39^. For a zone $Z_z$ with *n* witnesses, typical Byzantine fault tolerance requires:

$$\begin{aligned} n \ge 3f + 1 \ , \end{aligned}$$

where *f* is the maximum number of Byzantine witnesses that can be tolerated while maintaining correct consensus ^39–41^. This theoretical requirement aligns with the geometric constraints of establishing an *N*-dimensional zone with Byzantine fault tolerance. For instance, in a 2-dimensional space, a fault-tolerant zone definition requires a minimum of $N + 2 = 4$ coplanar witnesses to both define the region’s boundaries and tolerate one Byzantine witness. If one witness is faulty, the remaining three witnesses can still form a sufficient configuration to define a 2-dimensional zone. This minimal 4-witness, 2-dimensional zone structure is actually sufficient for most mapping and geolocation scenarios. Nonetheless, the number and coplanarity of witnesses can be increased or redefined, as needed, to flexibly enhance the fault tolerance of the system or define zones in higher-dimensional spaces.

**Fig. 4:**
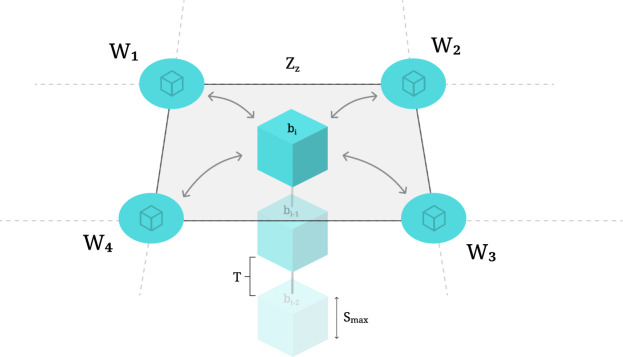
Deterministic finality Byzantine fault-tolerant consensus mechanism, in a non-hierarchical fully connected mesh network topology, resulting in a blockchain structure within a zone $Z_z$, where each block $b_i$ is content-bounded by maximum size $S_{\text {max}}$ and is generated on average every *T* time units.

Altogether, such a consensus mechanism then enables establishing, within the zone, a blockchain structure *B*:

$$\begin{aligned} B = \{b_0, b_1, ..., b_k\}, \text { where } \vert b_i \vert \le S_{max} \ , \end{aligned}$$

where each block $b_i$ is content-bounded by maximum size $S_{\text {max}}$ and is generated on average every *T* time units^41^. A blockchain is a distributed ledger that records data in a sequence of cryptographically linked blocks, maintained by a distributed network of participants^41^. This structure serves as the foundation for temporal synchronization of a bounded set of events, occurring within the zone (see Fig. 4). The regular block generation interval *T* establishes a logical clock that all witnesses use to coordinate their activities. For clarity, each block $b_i$ typically encapsulates the events recorded during its interval, such as the location proofs, digitally signed by the witnesses, and a timestamp marking the block’s creation. The next subsection goes into more detail on the content of the blocks, specifically regarding the location proofs. In practice, blocks are constructed by aggregating these inputs over the fixed interval *T*, and each block includes a cryptographic hash linking it to the previous block. This linkage ensures both the immutability and verifiability of the recorded data. Block validation is achieved via the Byzantine fault-tolerant consensus algorithm, which guarantees that only blocks reflecting the collective agreement of a sufficient number of witnesses are appended to the chain. These mechanisms collectively ensure that the data-comprising location proofs and metadata-remains trustworthy and tamper-resistant even in adversarial settings. Through the sequential nature of the blockchain, the sets of events within the zone are totally ordered, creating a consistent view of their temporal relationship.

Ultimately, the witnesses maintain consensus through a Byzantine fault-tolerant algorithm that ensures:

$$\begin{aligned} \forall w_i, w_j \in Z_z : t_{w_i} = t_{w_j} \Rightarrow \text {View}_{w_i}(B) = \text {View}_{w_j}(B) \ , \end{aligned}$$

where $\text {View}_w(B)$ represents a witness’s local view of the blockchain state. This strong consistency guarantee is essential for maintaining reliable temporal synchronization within the zone. Moreover, the consensus mechanism can be augmented with Turing-complete computational capabilities^39,42^, enabling the execution of arbitrary logic within the zone. This feature extends the basic consensus functionality to support complex zone-relative computations and verifications. Witnesses can execute and verify attestation rules, implement programmable location-based services, and even adjust zone parameters dynamically based on agreed conditions. The Turing-completeness property transforms the zone from a simple synchronized space into a programmable environment where complex location-based logic can be implemented and enforced collectively by the witnesses. The combination of Byzantine fault tolerance, deterministic finality, and Turing-completeness creates a foundation for secure programmable location attestation. This paper focuses on logical time synchronization, represented by the ordering of events within the blockchain. However, such a model can be extended to incorporate more precise temporal agreements^24,43^ and, eventually, global clock synchronization mechanisms for more advanced use cases.

### Relative PoL

To achieve relative PoL, we need a secure protocol capable of generating verifiable location proofs that can attest to the relative existence of a prover among a designated set of witnesses at a certain point in time. This system should build on the spatially bounded logical consensus mechanism outlined in the previous section, which allows witnesses to achieve unified agreements and align the timing of attestations for temporal coherence over the nearby presence of a prover. In brief, the protocol should enable the prover to generate a cryptographic proof that it was present within the specific zone $Z_z$ at a specific time instance, as agreed upon by the witnesses, given that the prover was in proximity to the witnesses in the zone at a moment in time equal for all of them. Therefore, the prover must physically interact with each witness in the zone to obtain valid shares of time attestation, which can be added together to form a collective proof, confirming to the verifier that the prover was indeed present within the zone at a time that the witnesses agreed upon.

Given the above, the protocol must ensure that the prover cannot forge a proof without physically interacting with the witnesses, and that the witnesses cannot collude to generate a false proof or prevent the prover from obtaining a valid proof. To achieve these goals, the protocol must rely on a set of cryptographic primitives that can ensure the security of the proof, while also providing a practical and efficient mechanism for the prover to interact with the witnesses. Among the cryptographic mechanisms that can be used to achieve these goals are distributed digital signature schemes^44^ and distance bounding protocols^45,46^. Distributed digital signature protocols, such as threshold or multi-signature schemes, can be used to distribute the attestation among the witnesses, ensuring that there is a minimum number of witnesses that can generate a valid proof. Distance bounding protocols rely on the speed of light to establish an upper bound on the distance between two entities and can be used to establish the physical proximity of the prover to the witnesses, preventing the prover from claiming proximity without being physically close. The witnesses may not yet make any contextual judgment about the validity of the prover’s claim; at this point, they may only attest to the fact that the prover was present within the zone at a specific time instance. The verification of the proof is left to the verifier, who can use the collective attestation of the witnesses to judge the prover’s claim. Nonetheless, in the future, more complex sensory attestations may be integrated to extend these location claims.

The following outlines the step-by-step execution of the relative PoL protocol, including its underlying assumptions and verification logic:

**Fig. 5:**
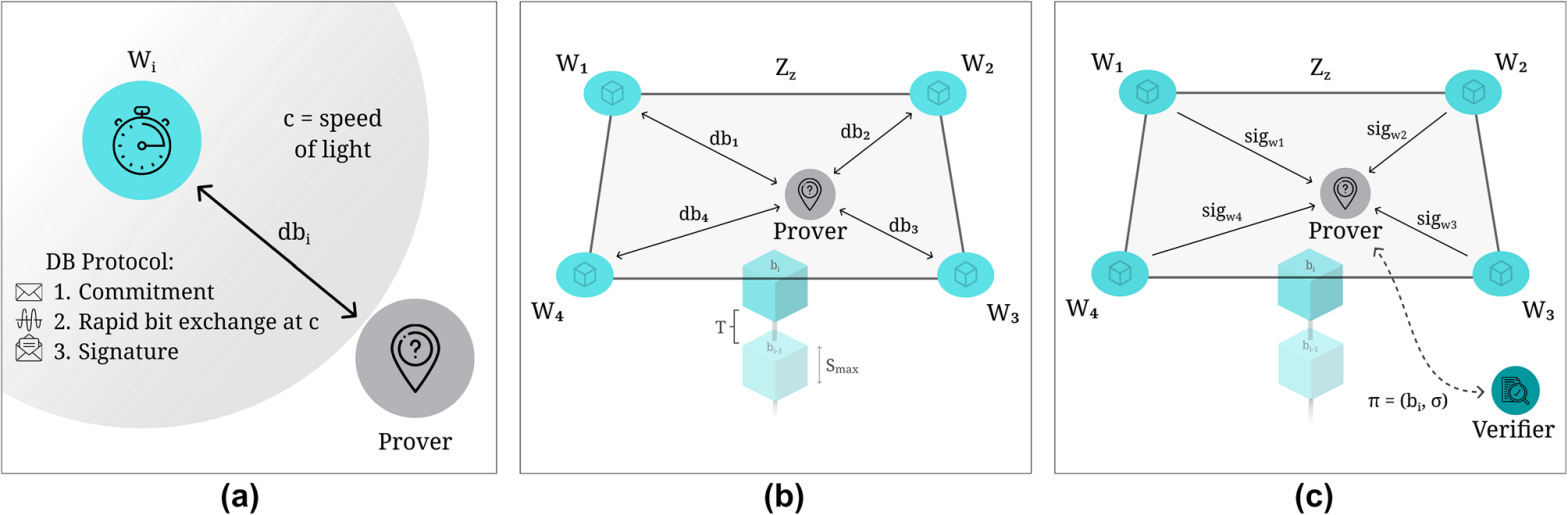
Relative PoL protocol within a zone $Z_z$, where the prover *p* generates a location proof by (a) interacting with each witness $w_1, w_2, \dots , w_{n}$ to (b) obtain individual distance bounds $db_1, db_2, \dots , db_{n}$ and (c) compile a collective signature $\sigma$ over the block $b_i$, to be later presented to the verifier.

**Preconditions.**

- Each witness $w_i \in Z_z$ possesses a public-private key pair $(pk_{w_i}, sk_{w_i})$ used to sign block contents.
- The prover *p* has a known identity and public key $pk_p$.
- A secure distance bounding protocol $\text {DB}(w_i, p)$ is available.
- Witnesses agree on a common block candidate $b_i$ containing prover data and metadata.
- A threshold signature scheme is used, requiring at least $n_b$ partial signatures (out of *n* total) to generate a valid collective signature $\sigma$, tolerating up to $n - n_b$ Byzantine witnesses.

**Protocol execution.** To initialize the protocol, the prover engages in a verifiable multilateration process^2,47^ with all the witnesses in zone $Z_z$:

1. **Distance bounding.** The prover *p* engages in a secure distance bounding protocol with each witness $w_i \in Z_z$, resulting in individual upper bounds:
  $$\begin{aligned} db _i = \text {DB}(w_i, p) \end{aligned}$$
2. **Partial signature generation.** Each witness $w_i$ computes a partial digital signature over the current block candidate $b_i$, which includes the prover identity, the distance bounds, and any additional metadata:
  $$\begin{aligned} \text {sig}_{w_i} = \text {Sign}_{sk_{w_i}}(b_i) \end{aligned}$$
3. **Proof collection.** The prover collects at least $n_b$ partial signatures via the channel $C_p$, forming the set:
  $$\begin{aligned} \{\text {sig}_{w_1}, \dots , \text {sig}_{w_{n_b}}\} \end{aligned}$$
  and constructs a collective signature:
  $$\begin{aligned} \sigma = \text {Aggregate}(\text {sig}_{w_1}, \dots , \text {sig}_{w_{n_b}}) \end{aligned}$$
4. **Proof assembly.** The prover assembles the final location proof:
  $$\begin{aligned} \pi = (b_i, \sigma ) \end{aligned}$$
  This proof is then submitted to a verifier, representing a collective attestation of the prover’s relative proximity to the zone $Z_z$ at a specific time.

**Verification.** Upon receiving the proof $\pi = (b_i, \sigma )$, a verifier can assess the validity of the prover’s claim through the following steps, detailed further in Algorithm 1, in the **Methods** section:

1. **Signature verification:** Confirm that $\sigma$ is a valid collective signature over $b_i$ and was generated by at least $n_b$ witnesses.
2. **Zone consistency check:** Extract $\{ db _i\}$ from $b_i$ and use the distance bounds and known witness positions to estimate the prover’s position via multilateration^2^, verifying that it lies within the zone defined by $Z_z$ or conforms to the required spatial constraints.

A schematic view of the interactions is provided in Fig. 5, and the full sequence is illustrated in Fig. 10. A security analysis is provided at the end of section **Discussion**.

As the protocol progresses, the prover and witnesses can continue to generate additional proofs, which are appended to consecutive blocks of the blockchain. For each new time instance, defined by the block interval *T*, the prover interacts with the witnesses in zone $Z_z$ to obtain updated distance bounds $db _1, db _2, \dots , db _n$ and initiate a fresh round of the protocol. This results in a new PoL claim associated with a new block $b_{i+1}$ which is partially signed by each witness. The block interval *T* determines the pacing of these proofs, providing a consistent temporal structure to the blockchain. Each interval *T* corresponds to a new block in the blockchain where a new PoL claim can be recorded, ensuring a regular cadence for updates in the prover’s location attestations and synchronization across witnesses within the zone. This structure ultimately extends the protocols in^2,5,45,47^ with a size-bounded, hash-linked time dimension. Additionally, the interval *T* defines the timeframe within which the prover must engage with all required witnesses in the zone to gather the necessary distance bounds for a valid proof. The more restricted it is, the less room it gives for possible attacks, but the more impractical it becomes for an honest prover. However, its asymptotic approximation to zero is what guarantees the theoretical correctness of such protocols^5^.

Moreover, this system allows multiple provers in the same zone $Z_z$ to simultaneously generate their own PoL claims. Each prover’s PoL claim is recorded independently, and the blockchain maintains a chronological ordering of all provers’ interactions with the witnesses. This serialized structure ensures that all events within the zone are preserved in a unified and ordered ledger, providing verifiers with a coherent timeline of presence proofs for multiple entities. By generating proofs for consecutive blocks, the protocol creates a sequential set of location proofs reflecting the temporal presence and movement of each prover within the zone. Each block added to the blockchain contains a snapshot of a prover’s location claim, validated by the witnesses, thereby establishing a serialized and tamper-resistant record of events within zone $Z_z$. This serialization of events is crucial for applications requiring an ordered record of location proofs, as it preserves the precise sequence of interactions within the blockchain. The granularity of time discretization within the zone is influenced by both the block interval *T* and the maximum block size $S_{\text {max}}$. Readjusting these parameters may impact the blockchain’s security and scalability, as well as the precision of time serialization required within the zone^39^.

### Absolute PoL

Building on the foundation of Relative PoL where space and time synchronization are maintained within a localized zone, the next logical step is to extend the protocol to enable Absolute PoL. Achieving this requires integrating the existing protocols with a Global Time Synchronization Protocol and a Global Positioning System. The objective is to generate a PoL certificate that is spatio-temporally sound within a local zone but can also be universally recognized outside the zone.

For temporal synchronization, Global Time Synchronizers can provide precise timestamps that are verifiable across the network. Protocols such as the Network Time Protocol (NTP) and Precision Time Protocol (PTP) are commonly used to synchronize time over distributed systems^43,48,49^. Its integration into the zone’s consensus mechanism can enable decentralized attestation of time, creating a shared global temporal framework that all witnesses can agree upon. Alternatively, blockchain-based time-stamping, as exemplified by systems like Bitcoin or Ethereum, can act as a decentralized global clock, embedding and anchoring time-stamped data within a public ledger to create verifiable and immutable zone-independent time records^50^. On the spatial front, Absolute PoL may similarly rely on the agreement over the positioning data from a Global Positioning System (GPS) or other positioning infrastructure, coupled with a universally agreed-upon geographic coordinate standard. Furthermore, incorporating Discrete Global Grid Systems (DGGS)^51^, such as Geohashing^24,52^ or Hexagonal Hierarchical Geospatial Indexing^53,54^, would allow for unique zone identification with consistent but flexible spatial granularity. DGGS methods help discretize the earth’s surface into polygonal cells, making it possible to assign each geographic area a unique identifier, which simplifies location verification and enhances mapping services interoperability^51,52^. The choice between these approaches may depend on trustworthiness requirements between the use case entities which should define their consensual time and space sources, standards, and protocols.

**Fig. 6:**
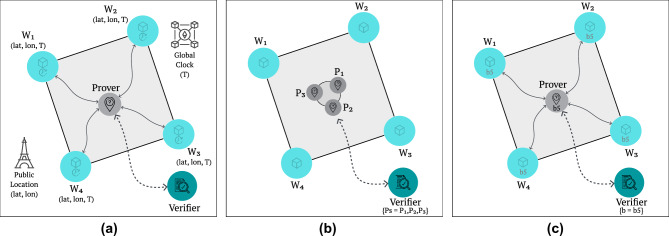
More sophisticated use cases such as (**a**) achieving Absolute PoL by combining Relative PoL with a Global Time Synchronization Protocol and a Global Positioning System, (**b**) a multi-prover configuration, or (**c**) the case where a verifier enforces the attestation of the prover’s location at a specific block or time.

The integration of Absolute PoL extends the capabilities of Relative PoL, potentially enabling programmable location-based services within each zone through Turing-complete functionalities, akin to Ethereum’s approach to smart contracts, as introduced in section **Zone Synchronization**. By embedding programmable logic within zones, Absolute PoL allows consensus-based, decentralized verification of location proofs that can adapt to more dynamic use cases. This system would support complex applications like those outlined in section **Application Scenarios**, where location proofs are not only verified but can trigger predefined actions or fulfil contractual obligations based on spatial and temporal conditions. For instance, location-based permissions, access controls, or rewards can be encoded as smart contracts that automatically execute when the required location conditions are met. Figure 6 presents some examples of this, such as a multi-prover configuration and verifiable attestations that tie a prover’s location to a specific time instance. Additionally, with global time synchronization in place, temporally consistent proofs can be generated for provers moving across zones in the latticework, ensuring continuous spatio-temporal attestation as they navigate through the network, as depicted in Fig. 2.

To realize the outlined applications, a digital identity infrastructure is essential. Every entity - provers, witnesses, and verifiers - must have a verified digital identity within the system. Witnesses, especially, need an identity association with their respective zones’ identity. This can be achieved by establishing secure digital identities tied to a Public Key Infrastructure (PKI) system, which would facilitate reliable authentication and secure communication among all parties^55,56^. If becoming part of a government or nation-wide infrastructure, the PoL system could leverage or extend existing PKI frameworks to ensure compliance with national identity and regulatory standards^57,58^.

Combining these elements enables the creation of Absolute PoL certificates. Such certificates could be universally accepted, allowing any entity, regardless of its location, to validate the spatio-temporal authenticity of a PoL claim. Nonetheless, this relative PoL architecture is flexible enough to support additional attributes, such as higher-level certificate formats, advanced trust settings, and composite information, paving the way for more applications and future enhancements in global location attestation.

### Emulation and evaluation

We did preliminary estimations and evaluations of the relative PoL protocol within a single zone to assess the system’s scalability, reliability, and efficiency, guiding deployment strategies and protocol design choices for future implementations. We created an emulation environment to assess one witnessing zone and relative PoL protocol, focusing on the witness count, network traffic, and synchronization dynamics. The emulation environment consisted of a set of virtual machines representing witnesses and provers, interconnected through a virtual network bridge where we simulated network constraints, such as topological connections, distance latency, or packet loss, with the witnesses forming a non-hierarchical fully connected mesh network topology. We assumed a city-block scenario where the witnesses were distributed across a 2-dimensional space, with $R_{\text {max}}$ around 100 meters, and the prover to be 50 meters away from each witness. The witnesses maintained a blockchain structure within the zone, with varying block interval *T*, and generated location proofs for a prover interacting with them. Section **Methods** provides a description of the emulation environment, architecture, and protocol settings.

**Fig. 7:**
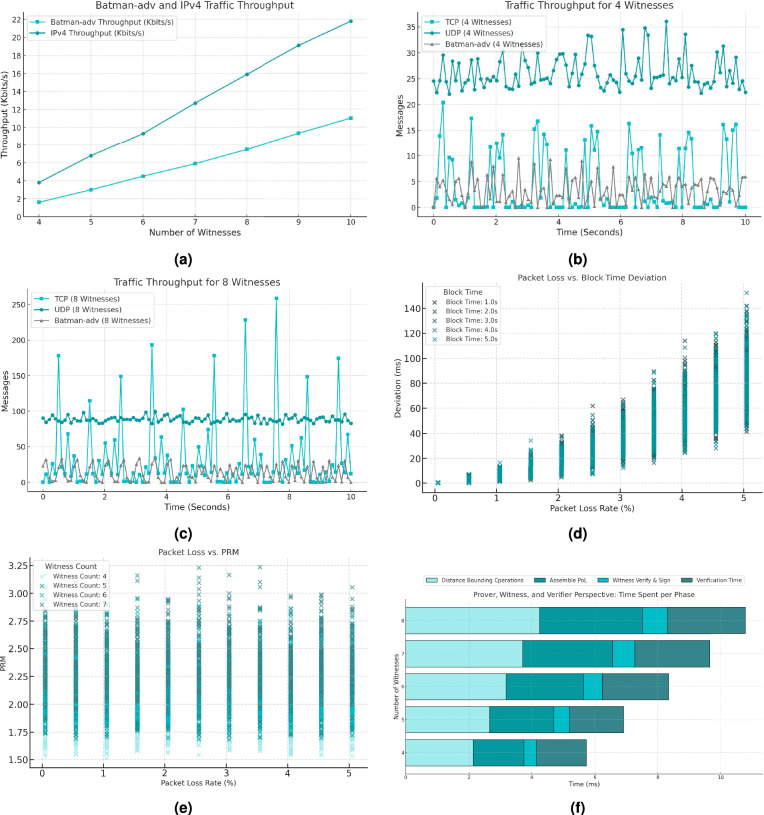
Average throughput for mesh (batman-adv) and network (IP) protocols (**a**), mesh and transport layer traffic patterns for 4 and 8 witnesses (**b** and **c**), deviation of the block generation time for a single witness in relation to packet loss (**d**), pairwise relative misalignment (PRM) for multiple witnesses in relation to packet loss (**e**), time spent in specific protocol phases, without accounting for communication delays and idle waiting times (**f**)

The bridge interface, which aggregates all mesh traffic exchanged between witness interfaces, served as the baseline for networking measurements. During the establishment of mesh connectivity within a zone, mesh protocol frames had an average size of 74 bytes, while network layer packets averaged 278 bytes. As illustrated in Fig. 7a, both protocols exhibited a linear increase in throughput as the number of instances grew. Additionally, blockchain activity was monitored to analyse protocol behaviour during block generation phases. Figures 7b and 7c depict the number of messages exchanged between instances over a typical protocol activity period. With the block interval set to 1 second, the peaks in transport layer traffic coincide with block generation events. As the number of instances increases, this effect becomes more pronounced; however, overall traffic remains proportionally low, with minimal impact on network performance.

From these observations, we further evaluated the protocol behaviour. Use cases requiring tighter temporal alignment and precise event ordering within zones must carefully balance block interval selection and network stability. Specifically, reducing the block interval can enhance alignment or at least minimize misalignment errors by committing location proofs more often; however, such configurations demand environments with low network disturbances. Delays related to distance may have no major impact on the protocol in such small zones, but if disturbances like packet loss are not mitigated, they can compound over time, significantly degrading the temporal precision of location proofs, especially in real-time or high-cadence scenarios. For a single witness (Fig. 7d), this misalignment may result in delays when deciding in which block to commit its captured distance bound. If not deciding in agreement with the rest of the witnesses, such delays can reach the size of an entire block interval, introducing substantial timing variability in proof generation. Moreover, as the number of witnesses increases, the pairwise relative misalignment (PRM) grows with greater variability (Fig. 7e):

$$\begin{aligned} PRM = \frac{T_{\text {latest}} - T_{\text {earliest}}}{\frac{\sum _{i < j} |T_i - T_j |}{C(W, 2)}} \ , \end{aligned}$$

where $T_{\text {latest}}$ and $T_{\text {earliest}}$ represent the latest and earliest measured local timestamps among witnesses, while the denominator normalizes the misalignment relative to the average pairwise timing differences. $PRM > 1$ indicates that the latest and earliest timestamps are further apart than the average pairwise differences, suggesting increased variability in witness synchronization. This variability stems from the compounded synchronization delays associated with increased communication complexity, which can affect the witness agreement threshold required to generate a valid proof for the prover. Thus, in zones with higher witness counts, under increasing packet loss, PRM variability may undermine alignment, necessitating coordination mechanisms to ensure timely and consistent proof generation. Witnesses operating in higher packet loss environments must account for these alignment challenges, potentially implementing mechanisms to coordinate requesting and committing specific location proofs to specific blocks. Controlling $R_{\text {max}}$, choosing between wired or wireless physical communication, and the number of witnesses in a zone can also help mitigate these issues, as smaller zones with fewer witnesses can reduce the complexity of witness interactions and improve synchronization.

The efficiency of proof generation in the protocol is influenced by the number of witnesses required for a valid proof, as increasing witness counts proportionally raise the prover’s overhead for collecting distance bounds and signatures (Fig. 7f). Each witness interaction introduces communication and processing delays, which can accumulate. In the worst-case scenario, a misaligned proof request may need to wait an entire block interval to collect signatures, significantly impacting total generation time. The number of proofs that can fit within a block interval depends on the ability to parallelize requests and the witnesses’ capacity to handle multiple interactions, constrained by block size. Verification of location proofs, however, may be relatively faster, requiring only validation of the collective signature and calculation of the prover’s position, which can be performed in parallel. Our evaluations in the emulation environment suggest that total proof generation will depend on factors such as prover-witness interaction time, witness count, and communication efficiency. Regardless of individual prover-witness interaction speed, proofs can only be finalized and submitted at the end of the block interval.

Furthermore, the experiments reported here were restricted to a single zone and single-prover-multi-witness scenarios. This focus reflects two complementary considerations. First, a single zone constitutes the minimal unit of spatio-temporal consensus and provides the essential foundation for understanding protocol behaviour before extending to more complex configurations. Second, while real-world deployments may involve overlapping or interconnected zones, supporting such configurations introduces distinct challenges such as inter-zone synchronization, zone affinity management, and scalable routing across zones, which are not yet adequately addressed in the literature. Existing protocols do not adequately meet these requirements, therefore, further research is needed on novel mesh networking algorithms to maintain consensus and communication in evolving witness topologies, which is beyond this evaluation’s scope.

At the same time, multiple provers operating concurrently within a single zone introduce scalability pressures related to network load and proof finalization time. This choice reflects an engineering trade-off in real-world implementations. Notably, distance bounding and threshold signature collection must both finalize within one block interval. Data from Fig. 7d and 7f indicate that one interval may admit hundreds of parallel proofs, though block size remains a fundamental scalability constraint, which relates to the blockchain trilemma^41^. Future work will explore these scalability limits in both multi-prover and multi-zone configurations, including overlapping zone interactions and composable location claims.

Further analysis involved continuous monitoring of both CPU and RAM usage throughout the experiments. On average, CPU utilization remained at 2% across the two virtual cores of each instance, spiking to 20% during prover-witness interactions. RAM consumption remained consistently low, never exceeding 200 MB. These findings align with expectations for the lightweight consensus mechanism, confirming its suitability for resource-constrained environments. However, these estimates depend on network conditions, processing capabilities, and implementation details. Future evaluations should involve optimized protocol implementations, real-world deployments, and adversarial scenarios to assess scalability and efficiency, including a breakdown of processing times and network overhead to identify and address performance bottlenecks.

## Discussion

Developing and deploying a decentralized PoL system requires careful consideration of practical aspects, particularly the hardware, networking, and cryptographic technologies involved, along with their implications on privacy and scalability.

### Cryptography foundations and privacy enhancements

On the cryptographic front, the PoL system relies on provably secure cryptographic mechanisms that are well-understood and widely adopted in various security-sensitive applications. These mechanisms form the foundation for further enhancements, such as privacy-preserving techniques. Research in selective disclosure protocols has demonstrated the feasibility of enabling provers to disclose only necessary location information, protecting sensitive data while generating verifiable proofs^3,22,26,36,53^. Building on this foundation, Zero-Knowledge Proof-of-Location (ZK-PoL) represents a promising path in the literature^22,26,53^, offering protocols that allow provers to demonstrate compliance with location requirements while preserving privacy by not revealing their exact position. Secure multi-party computation (MPC) is another cryptographic technique that has been explored, enabling multiple parties to jointly compute a function over their inputs without revealing them. MPC could be used, for example, at the witness level to jointly compute the prover’s location without revealing the individual distance bounds^5,10^. ZKPs applied at the network level is also a new and promising area of research, allowing for the verification of network properties without revealing sensitive information^59^.

### Secure communication and proximity technologies

Mesh networking technology, central to the communication backbone of such a system, has become ubiquitous within modern networking stacks and is widely adopted in consumer applications such as smart homes, sensor networks, and autonomous machines^37,60^. Major physical layer protocols like LoRa, WiFi, and Bluetooth provide upper-layer mesh networking solutions, making it easier to create self-organizing and fault-tolerant networks with many-to-many communication capabilities, for different ranges and data rates^37^. For instance, IEEE 802.11s, an amendment to the WiFi standard, enables Layer 2 (the OSI Model Data Link Layer) mesh networking with dynamic link discovery and maintenance through beacon transmissions^61^, while supporting routing protocols like the Hybrid Wireless Mesh Protocol (HWMP)^62^ or B.A.T.M.A.N.^63,64^. Bluetooth, on the other hand, is evolving to support mesh networking in consumer-grade devices, particularly with its upcoming Bluetooth 6 standard, which promises enhanced range, energy efficiency, and support for many-to-many connections^65^. These advances make mesh networking a viable solution for establishing resilient, flexible communication structures. Nonetheless, future research should focus on optimizing mesh networking for low-latency, high-throughput, and energy-efficient operations, essential for real-time location proofs. Additionally, none of the existing mesh networking protocols provide efficient support for managing interactions between overlapping zones-such as witness participation across zones, boundary synchronization, and inter-zone coordination. These remain largely unexplored and represent important challenges for future research.

In addition to mesh networking, distance bounding protocols play a crucial role in providing proximity verification between witnesses and provers. Distance bounding protocols are already employed in numerous applications, such as NFC, RFID systems, contactless cards, and access control UWB tags, to ensure secure and accurate distance measurements^46^. Practical implementations of these protocols, however, often require specific hardware and electronics integration to maintain accuracy. Nonetheless, the Bluetooth 6 standard is anticipated to incorporate distance bounding functionality, potentially bringing sub-meter accuracy for measurements at distances up to 150 meters to be achieved with consumer electronics^66^. The distances allowed by distance bounding protocol implementations will dictate the granularity of the zones in the PoL system, which can range from a few meters to hundreds of meters, depending on the application requirements. Reported implementations of distance bounding protocols, such as the challenge reflection with channel selection (CRCS), achieve accuracies up to 0.15 meters under ideal conditions with processing times as low as one nanosecond^67,68^. Further research is needed to adapt these protocols to noisier or more obstructed environments and maintain acceptable accuracy. We also expect protocol vendors to offer real-world performance insights that advance their practical deployment. Nevertheless, practical deployments must account for real-world factors like signal multipath propagation, obstructions, and processing delays, which may impact the achievable accuracy. Most distance bounding protocols involve analog-to-digital conversions that introduce additional processing delays, and practical accuracies are often closer to tens of meters in challenging environments^67^. Combining distance bounding protocols with consensus and mesh networking technologies is another area of research that could enhance the accuracy and security of location proofs.

While current distance bounding protocols focus on prover-witness proximity, our architecture also assumes that communication between witnesses is confined to physical proximity, constrained by the communication range $R_{max}$. However, in open or permissionless deployments, such as public urban environments with permissionless consensus mechanisms, this assumption may no longer hold. Future protocols may need to include mechanisms to securely establish and verify the validity of witness-to-witness links, especially in settings without prior trust relationships. Potential directions include the use of cryptographic distance bounding between witnesses^69^, or physical-layer attestations that bind communication attempts to spatial constraints. These enhancements would be essential for preventing long-range link spoofing, Sybil-based zone inflation, or collusion in trustless environments.

### Emerging applications and cross-domain extensions

An emerging and timely application domain for decentralized PoL systems is the authentication of physical-world content in the face of generative AI manipulation. As synthetic media becomes increasingly indistinguishable from genuine sensor data, it becomes essential to anchor digital content to verifiable spatio-temporal contexts^70^. Decentralized PoL infrastructure, when combined with sensor-based attestations, offers a promising foundation for such content authentication. Beyond simply proving presence at a location, future protocols could enable witnesses to capture complementary sensor data-such as environmental features, visual signatures, or ambient audio-and jointly attest to the physical conditions surrounding the prover at a given moment. These attestations could then be aggregated and cryptographically signed, yielding a multi-perspective proof that the claimed event or content was indeed grounded in physical reality^71^.

Achieving this would require extending the PoL model to support trust-weighted sensor fusion, where witness contributions are evaluated not only for spatial proximity but also for consistency and credibility across heterogeneous sensor modalities. It would also necessitate the development of content-aware verification layers capable of reasoning about temporal alignment, sensor diversity, and integrity under adversarial conditions. We view this direction as a crucial and multidisciplinary frontier, bridging location verification, sensor trust, and media integrity, and identify it as a compelling focus for future work.

### Security guarantees and threat model

We now provide an outline of the security guarantees of the proposed PoL system under a bounded adversarial model, focusing on its core design within a single-zone configuration.

**Threat model.** We consider a decentralized setting with potentially malicious provers and a subset of Byzantine witnesses, bounded by standard assumptions: in any zone, at most *f* out of *n* witnesses may behave arbitrarily, with $n \ge 3f + 1$. Witnesses are assumed to be operationally independent and equipped with hardware capable of executing distance bounding protocols. Communication is authenticated, delay-bounded, and subject to physical proximity constraints. In particular, links between witnesses (i.e., elements of $C_w$) are only established when nodes are within mutual communication range $R_{\text {max}}$. This confines zone formation to physically co-located witnesses and excludes long-range virtual link spoofing. Verifiers are honest but untrusted, and provers may attempt to forge proofs, misreport their position, or manipulate witness responses within the bounds of their physical and cryptographic capabilities.

**Security properties.** The system aims to satisfy the following two core spatio-temporal properties for each location proof:

- **Completeness:** If an honest prover *p* is physically present within a zone $Z_z$ at time $b_i$, and interacts with at least $n - f$ honest witnesses, then it can obtain a valid, verifiable location proof.
- **Soundness:** A malicious prover cannot obtain a valid location proof for a zone and time in which it was not physically present, except with negligible probability.

This security analysis is scoped to a single-zone setting, where the prover interacts with a quorum of co-located witnesses within one synchronized zone. Security guarantees in multi-zone deployments, including inter-zone coordination and proof composition, are left to future work.

**Mechanisms ensuring security.** These properties are enforced by the composition of protocol components:

1. **Distance bounding:** Enforces physical proximity through speed-of-light round-trip timing constraints. This prevents a remote prover from appearing closer than it is^46^.
2. **Witness quorum and signature aggregation:** Location proofs require threshold signature aggregation from a subset of $n_b$ honest witnesses, which must contribute, preventing proof generation by compromised majorities^44^.
3. **Multilateration and synchronization:** Multiple simultaneous distance bounds enable verifiable triangulation of the prover’s location^5,47^. When bounded within the same block interval, this guarantees that the location claim reflects a consistent, bounded region in both space and time. This setup mitigates relay attacks and enforces spatio-temporal cohesion, even in the presence of partial witness compromise.
4. **Blockchain consensus:** The witness blockchain provides tamper-evident ordering and finality of recorded events, protecting against temporal manipulation, replay attacks, and equivocation across zones^41^.
5. **Trust distribution:** Witness diversity across organizations and implementations mitigates correlated failures and adversarial collusion^72^, enhancing the resilience of the quorum.

**Security reduction.** Attacks against the system would require breaking one or more of the following primitives: (1) the security of the distance bounding protocol (e.g., via relay or timing attacks), (2) the unforgeability of the signature scheme, or (3) the safety and liveness of the underlying blockchain consensus protocol. A formal proof of soundness and completeness under this threat model could be constructed by reduction to these primitives.

Building on this foundation, we emphasize that this work proposes a system-level security model for decentralized PoL infrastructures. Rather than prescribing a fixed protocol instantiation, we outline how composable security guarantees can be achieved by combining well-defined cryptographic and physical-layer primitives. Full formal proofs for specific instantiations are left to future work, but the model defines how such instantiations can inherit security by construction.

As argued, within this compositional framework, the security of the relative PoL protocol relies on the robustness of its three core components: the blockchain consensus layer, the distributed signature scheme, and the distance bounding protocol.

Standardized distributed signature schemes exist and have been extensively studied^44^, ensuring that the witnesses can collectively produce a valid signature, for example, without compromising the security of a shared private key, in the case of typical threshold schemes. Other distributed schemes can be used to achieve the same goal^44,73,74^, depending on other specific requirements of the system, such as witness accountability or secure key management.

Similarly, distance bounding protocols have been developed to establish the physical proximity of two parties, and many of these protocols have been proven secure under various known adversarial models such as distance, mafia, or terrorist frauds^45,46,67,69^. Nonetheless, research is still ongoing to improve the practicality, efficiency, or privacy of these protocols, in the context of wireless communication and constrained devices.

Regarding untackled issues, verifiable multilateration still lacks a definitive solution for the case of an adversarial prover that may try to deceive the witnesses by deploying multiple synchronized entities and interacting with different witnesses simultaneously. Forcing the prover to conduct simultaneous and aggregated interactions with all witnesses is one way to mitigate this issue, but this solution reduces the protocol’s practicality^47^. A line of research on Position based Cryptography (PBC)^5,45,75^ has been developed to address this issue and supply the problem with a theoretical basis. The work shows that secure positioning is impossible in the vanilla model when adversaries deploying multiple devices have unbounded computational power, but can be achieved under other security models, namely when the attackers have bounded retrieval capabilities^5^. However, the practical implementation of such solutions is still an open challenge due to the initial assumptions on instantaneous communication and computations and perfect synchronization, which are not as feasible in real-world scenarios.

Despite these challenges, an important strength of our approach is its modularity. The relative PoL protocol is not bound to any specific cryptographic primitive; it can accommodate different signature schemes, distance bounding protocols, or consensus backends depending on the system’s trust model, decentralization goals, available hardware, and communication constraints-without altering the core verification logic.

### Comparison with existing PoL models and summary of contributions

Our proposed system is evaluated through a single-zone emulation, but it is important to situate it within the broader landscape of PoL architectures. Table 1 summarizes the conceptual evolution from centralized to fully decentralized PoL systems (see also **Related Work**), focusing on trust assumptions, synchronization requirements, and core security guarantees.

**Table 1:** Evolution of PoL architectures and their guarantees.

Architecture	Trust Assumptions	Synchronization Model	Security Guarantees and Limitations
Centralized^1,3,6,15–17^	Fully trusted server or location manager. Often assumes trusted client hardware.	Timestamp and location reference controlled by central entity.	Authenticity and basic integrity. Vulnerable to single-point failure. Lacks collusion and spoofing resistance, but suitable for closed or centrally governed environments.
Partially Distributed^10,16,18–21^	Small set of known anchors or CA-backed witnesses. May rely on secure sensor hardware or trusted time and location sources.	Local or witness-level timestamping. Often no global consensus.	Improved fault tolerance. Limited decentralization and composability. Often lacks enforceable spatio-temporal soundness or tamper resistance.
Decentralized (this work)^13,14,22,24,25^	Witness set with threshold-based trust; no single authority. No trusted hardware assumptions.	Consensus-based ordering, blockchain-backed time reference, local multilateration.	Spatio-temporal soundness. Resistance to collusion and tampering. Modular, composable architecture for permissionless deployment.

While several distributed PoL systems have been proposed, including privacy-preserving and consensus-based designs, the most relevant and actual decentralized systems, such as FOAM^24^, remain commercial, unpublished, or lack reproducible evaluation pipelines. We therefore refrain from quantitative comparisons, as existing proposals differ significantly in scope, threat model, architectural assumptions, and evaluation methodology. We also note that, while centralized architectures offer limited security guarantees in open environments, they may still be effective in tightly controlled or centrally governed deployments where trust boundaries are clearly defined. Rather than aiming to outperform prior work on a specific metric, our goal is to formalize a fully decentralized PoL framework grounded in composable cryptographic guarantees for spatio-temporal soundness and completeness.

**Summary of contributions.** To our knowledge, this is the first work to:

- Propose a system-level model for decentralized PoL systems based on a latticework of fault-tolerant witnessing zones.
- Introduce a structured model for spatio-temporal synchronization within zones, with support for multi-zone expansion.
- Formalize spatio-temporal soundness and completeness as core properties via a compositional model that integrates consensus, distributed digital signatures, and distance bounding protocols.
- Implement and evaluate the core system in an emulated environment, analyzing scalability, latency, and temporal alignment under varying witness counts.
- Outline and contextualize a diverse range of PoL use cases-including civic participation, content authentication, logistics, infrastructure auditing, and urban mobility-arguing for the need for a unified, general-purpose decentralized PoL system.

Many of the cryptographic properties we build upon-such as distance bounding, distributed digital signatures, and decentralized timestamping-have been addressed in isolation across prior work, but have not been integrated into a unified, fault-tolerant, and fully decentralized PoL model. To our knowledge, this is the first proposal to combine these guarantees under a structured architectural formalization. We believe this establishes a foundation for future work on multi-zone coordination, programmable location semantics, and advanced privacy-preserving layers.

## Methods

### Architecture and protocol integration

**Fig. 8:**
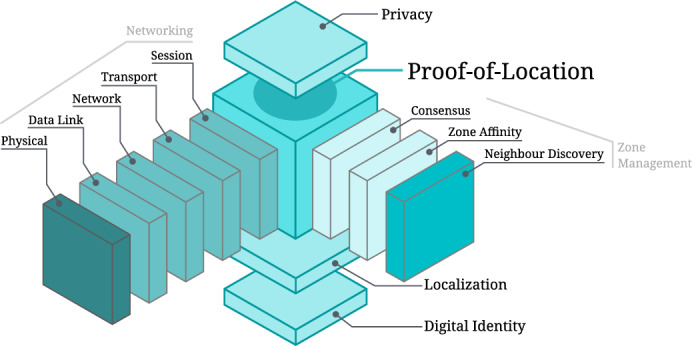
Layered architecture of a decentralized PoL system with the integration of different protocols and technologies^76^.

The proposed PoL system is designed as a layered architecture supporting the integration of various protocols and technologies to achieve a decentralized and scalable infrastructure. The system consists of six layers, illustrated in Fig. 8: the identity layer, localization layer, zone management layer, network layer, PoL layer, and privacy layer. Each provides specific functionalities and integration points for different protocol components. This architecture allows progressive and flexible improvements in scalability, reliability, security, and privacy. However, realizing a fully operational latticework infrastructure requires more than single-zone deployments. Multi-zone management introduces new challenges, including algorithms and protocols for zone discovery, neighborhood mapping, multi-zone affinity, and inter-zone synchronization. Enhancing techniques such as zone flooding and routing mechanisms may be essential to maintain consistent state across zones, particularly as provers move between them. These aspects define a new frontier in PoL scalability, as the infrastructure must support seamless transitions and continuous attestation across interconnected zones.

While current technologies provide a foundation for decentralized PoL, further progress in mesh networking, distance bounding accuracy, and privacy-preserving cryptographic techniques is essential for achieving scalable, real-world deployments. The envisioned multi-zone latticework requires continued research into practical implementation, interoperability, and privacy to address the challenges inherent in large-scale decentralized systems.

### Zone emulation and simulation

To evaluate the proposed architecture, we emulated a witnessing zone and simulated the PoL protocol interactions. This simulation demonstrated the feasibility of the system design and provided a controlled environment to assess performance, security properties, and scalability, as well as to inform future physical deployments.

The emulation focused on replicating decentralized mesh networking and distributed consensus. Using QEMU, we created virtual machines (VMs) representing provers and witnesses within a virtualized network. Each VM ran a custom OpenWrt image configured with the B.A.T.M.A.N.-Adv kernel module for ad hoc mesh networking and Ethereum tooling for blockchain operations. Virtual machines connected via bridged tap interfaces, simulating real-world communication conditions through injected delay, packet loss, and bandwidth limits (10 Mbit/s). Network traffic was monitored with Wireshark, and custom scripts were used to extract metrics such as computational load, consensus synchronization, and messaging behaviour. VM deployment, role assignment, and consensus setup were automated using provisioning scripts. The experiments ran on a host with an AMD Ryzen 7 4700U processor, 16 GB RAM, and an x86-64 Linux OS. Each VM was allocated 1 GB RAM and 2 vCPUs to replicate embedded device constraints.

**Fig. 9:**
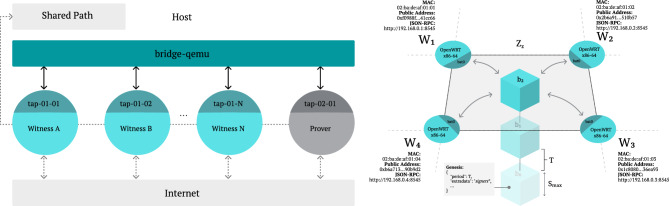
On the left, the network topology with witnesses and prover in an emulated witnessing zone. On the right, the simulated witnessing consensus mechanism with a PoA protocol.

Ethereum’s Proof-of-Authority (PoA) protocol was integrated to enforce temporal consistency and serialized transactions within the zone. Each witness acted as an authority node, signing blocks and validating transactions. PoA relied on a predefined signer list embedded in the genesis block^77^, and key generation was automated during initialization. Custom Golang tools, compiled for OpenWrt, handled genesis file creation, bootnode discovery, peer connectivity, and node lifecycle management. Nodes also exposed API endpoints for protocol interaction. To conserve resources, we enforced subnet-based communication restrictions and caching policies. Figure 9 illustrates the network topology and simulated consensus mechanism.

While the current implementation uses virtualization, future work aims to deploy the system on embedded devices with integrated distance bounding hardware to validate real-world feasibility. This will address hardware-specific challenges like physical zone management and network interface configurations, bridging the gap between simulation and field deployment.

### Proof generation and verification

**Fig. 10:**
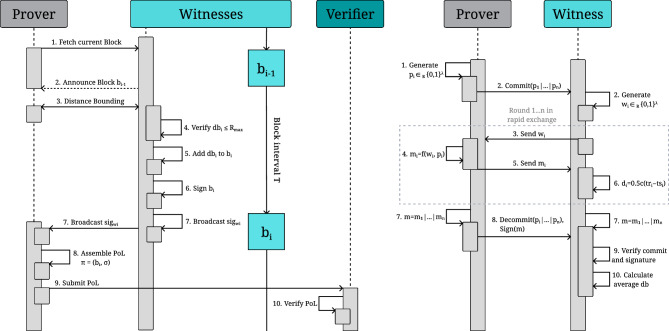
On the left, the sequence diagram of the proof generation process. On the right, standard distance bounding interactions between provers and witnesses^45^.

The proof generation process involves provers interacting with nearby witnesses to establish verifiable claims of presence. Distance bounding interactions and timings were simulated using estimates from related literature^45^, modeling realistic proximity bounds. Digital signatures were implemented with Ethereum tooling. Figure 10 illustrates the sequence of interactions between provers and witnesses. Verification follows generation, validating signatures and evaluating distance bounds via a multilateration algorithm. Described in Algorithm 1, and adapted from^2,47^, the method estimates the prover’s position using distance measurements and minimizes errors through the Minimum Mean Square Estimate (MMSE). It verifies geometric consistency using verification triangles formed by sets of three witnesses. The algorithm iterates over witnesses, computes distance errors, and minimizes the sum of squared errors. If the estimated position falls within any verification triangle, the location is accepted; otherwise, it is rejected.

While the proof-of-concept demonstrates feasibility, the protocols are designed for modularity. Different signature schemes and various identity infrastructures can be integrated, allowing adaptation to specific application needs and balancing security, performance, and privacy. Similarly, the multilateration algorithm can be extended to support tailored verifications and error correction. Future work will focus on refining these mechanisms to improve scalability, security, and privacy. Practical deployments may explore optimizations in signature aggregation, multilateration verification, and integration with privacy-preserving techniques such as zero-knowledge proofs or secure multiparty computation.

**Algorithm 1:**
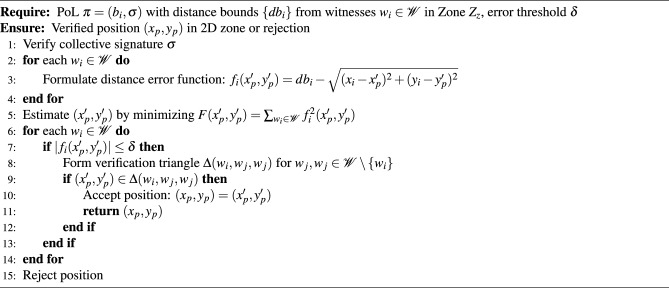
Verification algorithm^2,47^.

## Data Availability

The datasets generated from the experiments are available from the corresponding author on reasonable request.

## Code availability

The source code used in this study is publicly available at github.com/edurbrito-cyber/pol-emulation. It contains the portable components of the proof-of-concept emulation developed to evaluate the proposed system design. Please note that setting up the environment may require considerable effort, and the provided instructions are specific to certain host configurations. The manuscript includes the necessary context to assess the emulation setup and discusses relevant aspects of practical and real-world implementations.

## References

[1] Waters, B. & Felten, E. Secure, private proofs of location. *Department of Computer Science, Princeton University, Tech. Rep. TR-667-03* (2003).

[2] Capkun, S. & Hubaux, J.-P. Secure positioning in wireless networks. *IEEE Journal on Selected Areas in Communications* **24**, 221–232 (2006).

[3] Akand, M. R., Safavi-Naini, R., Kneppers, M., Giraud, M. & Lafourcade, P. Privacy-preserving proof-of-location with security against geo-tampering. *IEEE Transactions on Dependable and Secure Computing* (2021).

[4] King, R. J. Foam: The importance of time synchronization. https://blog.foam.space/foam-the-importance-of-time-synchronization-3934755ccc4e (2020). Accessed: 2024-11-02.

[5] Chandran, N., Goyal, V., Moriarty, R. & Ostrovsky, R. Position based cryptography. In *Annual International Cryptology Conference*, 391–407 (Springer, 2009).

[6] Saroiu, S. & Wolman, A. Enabling new mobile applications with location proofs. In *Proceedings of the 10th workshop on Mobile Computing Systems and Applications*, 1–6 (2009).

[7] Shu, K., Sliva, A., Wang, S., Tang, J. & Liu, H. Fake news detection on social media: A data mining perspective. *ACM SIGKDD explorations newsletter* **19**, 22–36 (2017).

[8] Yang, Z. *et al.* Group time-based one-time passwords and its application to efficient privacy-preserving proof of location. In *Annual Computer Security Applications Conference*, 497–512 (2021).

[9] Reichenbach, H. *The philosophy of space and time* (Courier Corporation, North Chelmsford, Massachusetts, USA, 2012).

[10] Dupin, A., Robert, J.-M. & Bidan, C. Location-proof system based on secure multi-party computations. In *Provable Security: 12th International Conference, ProvSec 2018, Jeju, South Korea, October 25-28, 2018, Proceedings*, 22–39 (Springer, 2018).

[11] Galison, P. *Einstein’s clocks and Poincaré’s maps: empires of time* (WW Norton & Company, New York, USA, 2004).

[12] Taylor, E. F. & Wheeler, J. A. *Spacetime physics* (Macmillan, London, UK, 1992).

[13] Amoretti, M., Brambilla, G., Medioli, F. & Zanichelli, F. Blockchain-based proof of location. In *2018 IEEE International Conference on Software Quality, Reliability and Security Companion (QRS-C)*, 146–153 (IEEE, 2018).

[14] Nasrulin, B., Muzammal, M. & Qu, Q. A robust spatio-temporal verification protocol for blockchain. In *Web Information Systems Engineering–WISE 2018: 19th International Conference, Dubai, United Arab Emirates, November 12-15, 2018, Proceedings, Part I 19*, 52–67 (Springer International Publishing, 2018).

[15] Graham, M. & Gray, D. Protecting privacy and securing the gathering of location proofs-the secure location verification proof gathering protocol. In *MobiSec*, 160–171 (Springer, 2009).

[16] Luo, W. & Hengartner, U. Veriplace: a privacy-aware location proof architecture. In *Proceedings of the 18th SIGSPATIAL International Conference on Advances in Geographic Information Systems*, 23–32 (2010).

[17] Javali, C., Revadigar, G., Rasmussen, K. B., Hu, W. & Jha, S. I am alice, i was in wonderland: secure location proof generation and verification protocol. In *2016 IEEE 41st conference on local computer networks (LCN)*, 477–485 (IEEE, 2016).

[18] Zhu, Z. & Cao, G. Applaus: A privacy-preserving location proof updating system for location-based services. In *2011 Proceedings IEEE INFOCOM*, 1889–1897 (IEEE, 2011).

[19] Wang, X., Pande, A., Zhu, J. & Mohapatra, P. Stamp: Enabling privacy-preserving location proofs for mobile users. *IEEE ACM Trans. Netw.* **24**, 3276–3289 (2016).

[20] Gambs, S., Killijian, M.-O., Roy, M. & Traoré, M. Props: A privacy-preserving location proof system. In *2014 IEEE 33rd International Symposium on Reliable Distributed Systems*, 1–10 (IEEE, 2014).

[21] Nosouhi, M. R., Yu, S., Grobler, M., Xiang, Y. & Zhu, Z. Sparse: privacy-aware and collusion resistant location proof generation and verification. In *2018 IEEE Global Communications Conference (GLOBECOM)*, 1–6 (IEEE, 2018).

[22] Wu, W., Liu, E., Gong, X. & Wang, R. Blockchain based zero-knowledge proof of location in iot. In *ICC 2020-2020 IEEE International Conference on Communications (ICC)*, 1–7 (IEEE, 2020).

[23] Nosouhi, M. R., Yu, S., Zhou, W., Grobler, M. & Keshtiar, H. Blockchain for secure location verification. *J. Parallel Distrib. Comput.* **136**, 40–51 (2020).

[24] Corp, F. Foam whitepaper. https://foam.space/publicAssets/FOAM_Whitepaper.pdf. Accessed: 2025-01-02.

[25] Pournaras, E. Proof of witness presence: Blockchain consensus for augmented democracy in smart cities. *J. Parallel Distrib. Comput.* **145**, 160–175 (2020).

[26] Li, W., Guo, H., Nejad, M. & Shen, C.-C. Privacy-preserving traffic management: A blockchain and zero-knowledge proof inspired approach. *IEEE Access* **8**, 181733–181743 (2020).

[27] Hancock, J. T. & Bailenson, J. N. The social impact of deepfakes (2021). 10.1089/cyber.2021.29208.jth 33760669

[28] Agarwal, S. *et al.* Protecting world leaders against deep fakes. In *CVPR workshops*, **1**, 38 (2019).

[29] Zhou, X. & Zafarani, R. A survey of fake news: Fundamental theories, detection methods, and opportunities. *ACM Computing Surveys (CSUR)* **53**, 1–40 (2020).

[30] Zafar, F., Khan, A., Anjum, A., Maple, C. & Shah, M. A. Location proof systems for smart internet of things: Requirements, taxonomy, and comparative analysis. *Electronics* **9**, 1776 (2020).

[31] Kaushik, V. & Suhas, P. Fool proof ticketing system for public transport. In *2018 3rd International Conference on Communication and Electronics Systems (ICCES)*, 56–60 (IEEE, 2018).

[32] Guo, J. et al. Tfl-dt: A trust evaluation scheme for federated learning in digital twin for mobile networks. *IEEE J. Sel. Areas Commun.* **41**, 3548–3560 (2023).

[33] Liu, Z. et al. Lightweight trustworthy message exchange in unmanned aerial vehicle networks. *IEEE trans. Intell. Transp. Syst.* **24**, 2144–2157 (2021).

[34] Liu, Z. et al. Tcemd: A trust cascading-based emergency message dissemination model in vanets. *IEEE Internet Things J.* **7**, 4028–4048 (2019).

[35] Boeira, F., Asplund, M. & Barcellos, M. Decentralized proof of location in vehicular ad hoc networks. *Computer Communications* **147**, 98–110 (2019).

[36] Li, W., Meese, C., Zhong, Z. G., Guo, H. & Nejad, M. Location-aware verification for autonomous truck platooning based on blockchain and zero-knowledge proof. In *2021 IEEE International Conference on Blockchain and Cryptocurrency (ICBC)*, 1–5 (IEEE, 2021).

[37] Cilfone, A., Davoli, L., Belli, L. & Ferrari, G. Wireless mesh networking: An iot-oriented perspective survey on relevant technologies. *Future Internet* **11**, 99 (2019).

[38] Sichitiu, M. L. Wireless mesh networks: opportunities and challenges. In *Proceedings of World Wireless Congress*, vol. 2, 21 (2005).

[39] Xiao, Y., Zhang, N., Lou, W. & Hou, Y. T. A survey of distributed consensus protocols for blockchain networks. *IEEE Commun. Surv. Tutorials* **22**, 1432–1465. 10.1109/COMST.2020.2969706 (2020).

[40] Castro, M., Liskov, B. *et al.* Practical byzantine fault tolerance. In *OsDI*, 173–186 (1999).

[41] Xiao, Y., Zhang, N., Li, J., Lou, W. & Hou, Y. T. Distributed consensus protocols and algorithms. *Blockchain for Distributed Syst. Secur.* **25**, 40 (2019).

[42] Buterin, V. et al. A next-generation smart contract and decentralized application platform. *white paper* **3**, 2–1 (2014).

[43] Malekpour, M. R. A self-stabilizing hybrid fault-tolerant synchronization protocol. In *2015 IEEE Aerospace Conference*, 1–11 (IEEE, 2015).

[44] Brandao, L. T., Mouha, N. W. & Vassilev, A. T. Threshold schemes for cryptographic primitives. *Natl. Inst. StandardsTechnol.*10.6028/NIST.IR.8214 (2019).

[45] Alexandru, A. Secure localization literature review. *Not peer-reviewed* (2021). Contains a list of peer-reviewed work on secure localization techniques.

[46] Akand, M. M. R. *Contribution to Proof-of-Location Systems.* Ph.D. thesis, University of Calgary, Alberta, Canada (2023).

[47] Chiang, J. T., Haas, J. J. & Hu, Y.-C. Secure and precise location verification using distance bounding and simultaneous multilateration. In *Proceedings of the second ACM conference on Wireless network security*, 181–192 (2009).

[48] Lévesque, M. & Tipper, D. A survey of clock synchronization over packet-switched networks. *IEEE Commun. Surv. Tutorials* **18**, 2926–2947. 10.1109/COMST.2016.2590438 (2016).

[49] Balakrishnan, K., Dhanalakshmi, R., Sinha, B. B. & Gopalakrishnan, R. Clock synchronization in industrial internet of things and potential works in precision time protocol: Review, challenges and future directions. *Int. J. Cogn. Comput. Eng.* **4**, 205–219 (2023).

[50] Konashevych, O. & Poblet, M. Blockchain anchoring of public registries: Options and challenges. In *Proceedings of the 12th International Conference on Theory and Practice of Electronic Governance*, 317–323 (2019).

[51] Li, M. & Stefanakis, E. Geospatial operations of discrete global grid systems-a comparison with traditional gis. *Journal of Geovisualization and Spatial Analysis* **4**, 26 (2020).

[52] Deiotte, R. & La Valley, R. Comparison of spatiotemporal mapping techniques for enormous etl and exploitation patterns. *ISPRS Annals of the Photogrammetry, Remote Sensing and Spatial Information Sciences* **4**, 7–13 (2017).

[53] Ernstberger, J., Zhang, C., Ciprian, L., Jovanovic, P. & Steinhorst, S. Zero-knowledge location privacy via accurate floating point snarks. arXiv preprint arXiv:2404.14983 (2024).

[54] Sahr, K. Central place indexing: Hierarchical linear indexing systems for mixed-aperture hexagonal discrete global grid systems. *Cartographica: The International Journal for Geographic Information and Geovisualization* **54**, 16–29 (2019).

[55] Adams, C. & Lloyd, S. *Understanding PKI: concepts, standards, and deployment considerations* (Addison-Wesley Professional, Boston, Massachusetts, USA, 2003).

[56] Stapleton, J. & Epstein, W. C. *Security without Obscurity: A Guide to PKI Operations* (CRC Press, Boca Raton, Florida, USA, 2024).

[57] Al-Khouri, A. M. Pki in government digital identity management systems. *European Journal of ePractice* **4** (2012).

[58] Wilson, S. The importance of pki today. *China Communications* (2005).

[59] Ernstberger, J., Chaliasos, S., Zhou, L., Jovanovic, P. & Gervais, A. Do you need a zero knowledge proof? *Cryptology ePrint Archive* (2024).

[60] Akyildiz, I. F., Wang, X. & Wang, W. Wireless mesh networks: a survey. *Computer networks* **47**, 445–487 (2005).

[61] Hiertz, G. R. *et al.* Ieee 802.11 s: the wlan mesh standard. *IEEE Wireless Communications* **17**, 104–111 (2010).

[62] Bari, S., Anwar, F. & Masud, M. Performance study of hybrid wireless mesh protocol (hwmp) for ieee 802.11 s wlan mesh networks. In *2012 international conference on computer and communication engineering (ICCCE)*, 712–716 (IEEE, 2012).

[63] Open-Mesh. B.a.t.m.a.n. v. https://www.open-mesh.org/projects/batman-adv/wiki/BATMAN_V. Accessed: 2024-12-21.

[64] Seither, D., König, A. & Hollick, M. Routing performance of wireless mesh networks: A practical evaluation of batman advanced. In *2011 IEEE 36th Conference on Local Computer Networks*, 897–904 (IEEE, 2011).

[65] SIG, B. Bluetooth mesh protocol 1.1. https://www.bluetooth.org/DocMan/handlers/DownloadDoc.ashx?doc_id=574298 (2024). Adopted Bluetooth specification defining fundamental requirements for an interoperable mesh networking solution for Bluetooth low energy wireless technology.

[66] Martin Woolley, B. S. Bluetooth channel sounding technical overview (2024). Bluetooth Channel Sounding Technical Overview by Martin Woolley, Bluetooth SIG.

[67] Akand, M. M. R. *In-region Location Verification Using Distance Bounding*. Ph.D. thesis, MS thesis. Graduate Studies (2016).

[68] Rasmussen, K. B. & Capkun, S. Realization of RF distance bounding. In *19th USENIX Security Symposium (USENIX Security 10)* (2010).

[69] Capkun, S., El Defrawy, K. & Tsudik, G. Group distance bounding protocols: (short paper). In *Trust and Trustworthy Computing: 4th International Conference, TRUST 2011, Pittsburgh, PA, USA, June 22-24, 2011. Proceedings 4*, 302–312 (Springer, 2011).

[70] The Coalition for Content Provenance and Authenticity (C2PA). The coalition for content provenance and authenticity (c2pa). https://c2pa.org/ (2022). Accessed on 2025-05-16.

[71] Castillo, F., Castillo, O., Brito, E. & Espinola, S. Trustworthy decentralized autonomous machines: A new paradigm in automation economy. arXiv preprint arXiv:2504.15676 (2025).

[72] Brito, E., Castillo, F., Pullonen-Raudvere, P. & Werner, S. Trustops: Continuously building trustworthy software. In *International Conference on Enterprise Design, Operations, and Computing*, 53–67 (Springer, 2024).

[73] Boneh, D., Partap, A. & Waters, B. Accountable multi-signatures with constant size public keys. *Cryptology ePrint Archive* (2023).

[74] Baum, C. et al. Efficient maliciously secure oblivious exponentiations. *Cryptology ePrint Archive* (2024).

[75] Zhang, J., Lu, N., Ma, J. & Yang, C. Universally composable secure geographic area verification without pre-shared secret. *Sci. China Life. Sci.* **62**, 1–15 (2019).30446870

[76] Peterson, L. L. & Davie, B. S. *Computer networks: a systems approach* (Morgan Kaufmann, San Francisco, USA, 2007).

[77] Proposals, E. I. Eip-225: Clique proof-of-authority consensus protocol. https://eips.ethereum.org/EIPS/eip-225 (2017). Accessed: 2024-12-17.

